# Metagenomic analysis of gut microbiota composition and function in wild mice (*Rattus flavipectus*) infected with *Enterocytozoon bieneusi*

**DOI:** 10.3389/fcimb.2025.1708266

**Published:** 2025-11-11

**Authors:** Hai-Long Yu, Rui Liu, Hai-Tao Wang, Qing-Yu Hou, Ya Qin, Xing Yang, Zhen-Qiu Gao, Li-Hua Yang, Quan Zhao, He Ma

**Affiliations:** 1 College of Veterinary Medicine, Jilin Agricultural University, Changchun, Jilin, China; 2 College of Life Sciences, Changchun Sci-Tech University, Shuangyang, Jilin, China; 3 College of Veterinary Medicine, Qingdao Agricultural University, Qingdao, Shandong, China; 4 Department of Medical Microbiology and Immunology, School of Basic Medicine, Dali University, Dali, Yunnan, China; 5 School of Pharmacy, Yancheng Teachers University, Yancheng, Jiangsu, China

**Keywords:** *Enterocytozoon bieneusi*, wild mice, gut virome, gut microbiota, function analysis

## Abstract

**Background:**

*Enterocytozoon bieneusi* (*E. bieneusi*) is a pathogenic microsporidian that infects a variety of hosts, including wild mice, potentially influencing their gut microbiota. This study aims to explore how *E. bieneusi* infection influences the gut microbiota composition and function in wild mice.

**Methods:**

Fecal samples were collected from 20 wild mice (*Rattus flavipectus*) in September 2023 in Yunnan Province, China. The PCR results showed that 10 were infected with *E. bieneusi* and 10 were uninfected, with no samples testing positive for *Cryptosporidium* spp.*, Blastocystis, Giardia, Cyclospora or Balantioides coli.* DNA was extracted and subjected to metagenomic sequencing using Illumina HiSeq. Gut microbiota composition was assessed using MetaPhlAn4 for species-level annotation. The contigs were used to construct a gene catalog and perform functional annotation. Additionally, viral sequences were identified by analyzing the contigs with software, such as CheckV and Vibrant.

**Results:**

The gut microbiota diversity showed no significant difference between mice infected with *E. bieneusi* and the control group, with the dominant phyla being *Firmicutes* and *Bacteroidetes*. Virome analysis identified 18,192 high-quality viral sequences, with the *E. bieneusi* group exhibiting higher viral species diversity. Furthermore, significant differences were observed in 178 viral operational taxonomic units (vOTUs) between the two groups, with 161 vOTUs enriched in the *E. bieneusi* group. Functional analysis demonstrated significant enrichment of several metabolic pathways in the gut microbiota of wild mice infected with *E. bieneusi*, particularly in the metabolism of terpenoids and polyketides, digestive system, biosynthesis of other secondary metabolites and metabolism of cofactors and vitamins. Notably, unique virus-bacteria correlations were observed in the *E. bieneusi* group.

**Conclusions:**

*E. bieneusi* infection significantly alters the gut virome in wild mice, affecting microbial composition and interactions. The infection appears to drive adaptive changes in microbial functions, especially in metabolic processes, suggesting a host response to infection-related stress.

## Introduction

The gut microbiota is essential in regulating various physiological processes, including immune system regulation, maintenance of metabolic homeostasis and defense against pathogens (Zheng et al., 2020; Li et al., 2024; Shang et al., 2025). Recent research has demonstrated that microbial communities are highly dynamic, with their diversity and function altered by pathogen infection (Meng et al., 2023). Among the pathogens that disrupt gut microbial balance, the microsporidian parasite *Enterocytozoon bieneusi* (*E. bieneusi*) has gained attention for its ability to infect diverse hosts, including humans and wild mice (Gao et al., 2024a).

Wild mice serve as significant hosts for *E. bieneusi*, playing an essential role in its transmission to humans and animals (Zhao et al., 2024). For instance, previous studies have shown that wild rats and shrews carry 33 *E. bieneusi* genotypes, with a 14.1% infection rate (Zhang et al., 2024). Similarly, *E. bieneusi* prevalence in wild mice across three Chinese provinces was found to be 11.92%, with *Rattus flavipectus* showing the highest rate (Gao et al., 2024a).

Although *E. bieneusi* is recognized as an important pathogen, its impact on the gut microbiota—particularly in wild mice—remains unexplored. Research on other infections, such as *Helicobacter pylori* and *Clostridium difficile*, indicated that these pathogens significantly change gut microbiome, highlighting the complexity of host-microbe interactions during infection (Gonzales-Luna et al., 2023; Li et al., 2024). Therefore, understanding how *E. bieneusi* infection affects the composition and function of the gut microbiota in wild mice is crucial for its public health implications.

The gut virome, a viral community in the gut, may interact with microbiota, affecting immune response and infection susceptibility (Tiamani et al., 2022). Although there is increasing acknowledgment of the significance of the virome, the majority of research continues to focus on gut microbiota, resulting in a lack of extensive research on the gut virome, particularly in wild animals. Research has shown that during pathogen infection, the viral community in the gut can undergo significant alterations, affecting microbiota relative abundance and even modulating host responses (Duerkop, 2018). However, the interaction between a eukaryotic gut parasite like *E. bieneusi* and the gut virome remains virtually unknown.

The central hypothesis of this study is that *E. bieneusi* infection may alter the gut virome, leading to significant shifts in viral diversity and functional pathways, which in turn could affect the overall gut microbial ecosystem. To explore this hypothesis, we analyzed metagenomic data from 10 samples of *E. bieneusi*-infected wild mice (*Rattus flavipectus*) and 10 uninfected samples to examine gut microbiota alteration. From the metagenomic samples, we identified 1,029 viral operational taxonomic units (vOTUs) and performed a comprehensive analysis of the gut virome. Notably, the gut virome composition differed significantly between the *E. bieneusi* and control groups, with 178 vOTUs exhibiting distinct relative abundances. Furthermore, our findings revealed a significant relationship between the gut virome and gut bacteria. Notably, we found that changes in the gut virome were more significant than those in the bacteriome.

## Methods

### Sample collection and DNA extraction

A total of 20 wild mice (*Rattus flavipectus*) fecal samples were collected in September 2023 in Yunnan Province, China. Wild mice were captured using mouse traps, and fecal samples were collected from the rectum of each mouse. The fecal samples were then placed in the boxes containing dry ice and transported to the laboratory, where they were stored at -20°C. DNA was extracted from the fecal samples using an Ezna Stool DNA Kit (Omega Biotek Inc., Norcross, GA, USA) according to the manufacturer’s protocol and stored at -20°C.

### PCR amplification, electrophoresis and metagenome sequencing

Among the 20 samples, 10 were infected with *E. bieneusi* and 10 were uninfected (Gao et al., 2024a), with no samples testing positive for *Cryptosporidium* spp., *Blastocystis*, or *Giardia infections* (Gao et al., 2024b; Gao et al., 2025; Qin et al., 2025). Meanwhile, we also performed PCR targeting *Cyclospora* and *Balantioides coli*.

For *Cyclospora*, the primers used for the first-round PCR were ExCycF (5′-AAT GTA AAA CCC TTC CAG AGT AAC-3′) and ExCycR (5′-GCA ATA ATC TAT CCC CAT CAC G-3′) (Relman et al., 1996). The PCR conditions were as follows: initial denaturation at 94°C for 7 minutes, followed by 35 cycles of 30 s at 94°C, 30s at 55°C, 90s at 72°C, with a final extension at 72°C for 7 minutes. For the secondary nested PCR, the primers NesCycF (5′-AAT TCC AGC TCC AAT AGT GTA T-3′) and NesCycR (5′-CAG GAG AAG CCA AGG TAG GCR TTT-3′) were used. For *Balantioides coli*, PCR was performed to amplify the ITS1-5.8S rRNA-ITS2 gene region using the primers B5D (5′-GCTCCTACCGATACCGGGT-3′) and B5RC (5′-GCGGGTCATCTTACTTGATTTC-3′) as described by the previous study (Li et al., 2020).

All PCR products were electrophoresed on 1% agarose gels, visualized under UV light. The sequencing libraries for the samples were prepared by Shanghai Personalbio Technology Co., Ltd and sequenced on the Illumina HiSeq platform with 150bp paired-end sequencing. The metagenomic samples were uploaded to the National Center for Biotechnology Information (NCBI) under the project accession number PRJNA1175865 (Shang et al., 2025).

### Pre-processing of sequencing reads

For quality control, raw metagenomic sequencing reads were processed with Fastp v0.23.0 (Chen et al., 2018), applying the parameters “-y -Y 30 -u 30 -q 20 -n 5 -l 80 –trim_poly_g.” Bowtie2 v2.5.0 (Langmead and Salzberg, 2012) was used to align sequences against the reference genome (NCBI RefSeq assembly GCA_036323735.1) with the parameters “–mm –end-to-end –fast” and to remove host-derived sequences. The remaining reads were regarded as clean reads. All samples were analyzed at the species level using MetaPhlAn4 v4.0.6 (Blanco-Míguez et al., 2023), with the following parameters “–nreads 20000000 -x mpa_vJan21_CHOCOPhlAnSGB_202103 –input_type sam.” Taxonomic unit relative abundance in the metagenomic samples was estimated through sequence similarity and the coverage of species-specific marker genes.

### Assembly and identification of viral sequences

Clean reads from each sample were assembled *de novo* using Megahit v1.2.9 (Li et al., 2015) with the k-mer parameter “-k-list 21, 41, 61, 81, 101, 121, 141.” Contigs longer than 5 kb were selected from each sample for viral sequence identification. These contigs were first processed with CheckV v1.0.1 (Nayfach et al., 2021) to exclude those containing more than 50% prokaryotic genes. The remaining sequences were designated as potential viral contigs based on any of the following criteria: (1) the presence of at least one viral gene and a higher count of viral genes than prokaryotic genes; (2) a DeepVirFinder score > 0.90 with a *p*-value < 0.01 (Ren et al., 2020); (3) classification as viral sequence by Vibrant v1.2.1 under default settings (Kieft et al., 2020).

In order to minimize non-viral contamination, we performed a decontamination procedure using the bacterial universal single-copy orthologs gene ratio (Busco v5.4.3) (Manni et al., 2021). Specifically, we computed the Busco gene-to-total gene ratio for each viral sequence and excluded sequences exhibiting a Busco ratio exceeding 5%.

### Viral clustering and taxonomic classification

To remove redundant viral sequences, pairwise comparisons were conducted using BLASTn v2.13.0 (Boratyn et al., 2013), and viruses with ≥95% nucleotide identity over at least 85% of their sequences were grouped into a vOTU. Sequence quality was evaluated using CheckV v1.0.1, while Vibrant v1.2.1 was performed to predict viral lifestyles. For taxonomic annotation, protein sequences were aligned with a comprehensive database, including Virus-Host DB (Mihara et al., 2016) and viral proteins from *Quimbyviridae*, *Gratiaviridae* (Benler et al., 2021), *Flandersviridae* and *crAss-like* (Guerin et al., 2018). Classification of a viral sequence into a family is based on over 25% of its proteins showing similarity to that family’s proteins.

### Taxonomic profiles

Taxonomic profiling of the gut virome was conducted by aligning clean reads from each sample against all vOTU reference sequences using Bowtie2 v2.5.0 with the “–no-unal –end-to-end –fast” parameters. To standardize sequencing depth across samples, mapped reads were randomly subsampled to 20 million reads per sample. The relative abundance of each vOTU was determined by normalizing the number of reads mapped to it by the total mapped reads. Relative abundances of all vOTUs classified under the same family were then summed to obtain the total abundance at the family level.

### Gene catalog and functional annotation

The contigs longer than 500 bp from 20 assembled samples were subjected to open reading frame (ORF) prediction using Prodigal v2.6.3 (Hyatt et al., 2010) in “meta” procedure. A non-redundant gene catalog with 10,345,443 ORFs was generated using MMseqs2 v7e2840992948ee89dcc336522dc98a74fe0adf00 (Steinegger and Söding, 2017) with the parameters “–cluster-mode 2 –cluster-reassign 1 –cov-mode 2 -c 0.9 –min-seq-id 0.95 –kmer-per-seq-scale 0.8 –kmer-per-seq 200.” Read counts (20 million reads) mapped to this catalog were then normalized to transcripts per kilobase million (TPM). Gene functional annotation was performed with DIAMOND v2.1.8.162 (Buchfink et al., 2015) against the Kyoto Encyclopedia of Genes and Genomes (KEGG) orthology (KO) and CAZy database, using the criteria “–min-score 60 –query-cover 50.”

### Statistical analysis and visualization

All statistical analyses were performed in R v4.2.2. Alpha diversity indices, including richness, simpson, and shannon, were calculated with the vegan v2.6.4. Beta diversity was assessed by computing Bray–Curtis distances between samples using the vegdist function from the same package. These distances were then subjected to principal coordinate analysis (PCoA) via the pcoa function. Additionally, PERMANOVA was conducted using the adonis function to evaluate group differences in microbial composition.

The rarefaction curve was generated using the vegan v2.6.4. The density plot was created using the ggpubr v0.6.0. The phylogenetic tree was visualized using iTOL v7.2.1. All other visualizations were generated using the ggplot2 v4.2.3.

### Identification of viral markers

Viral markers at the vOTU level were identified through the Wilcoxon test in the R stats v4.2.2 by comparing the *E. bieneusi* group with the control group, and *P* values were adjusted with p.adjust (stats v4.2.2), applying the “method = BH” option.

### Correlation analysis

The gut viral and bacterial marker correlation was assessed using cor.test with the “method = Spearman,” and data visualization was performed using the R ggraph v2.2.1.

## Results

### Gut microbiota composition and diversity in wild mice infected with *E. bieneusi*

**Figure 1:**
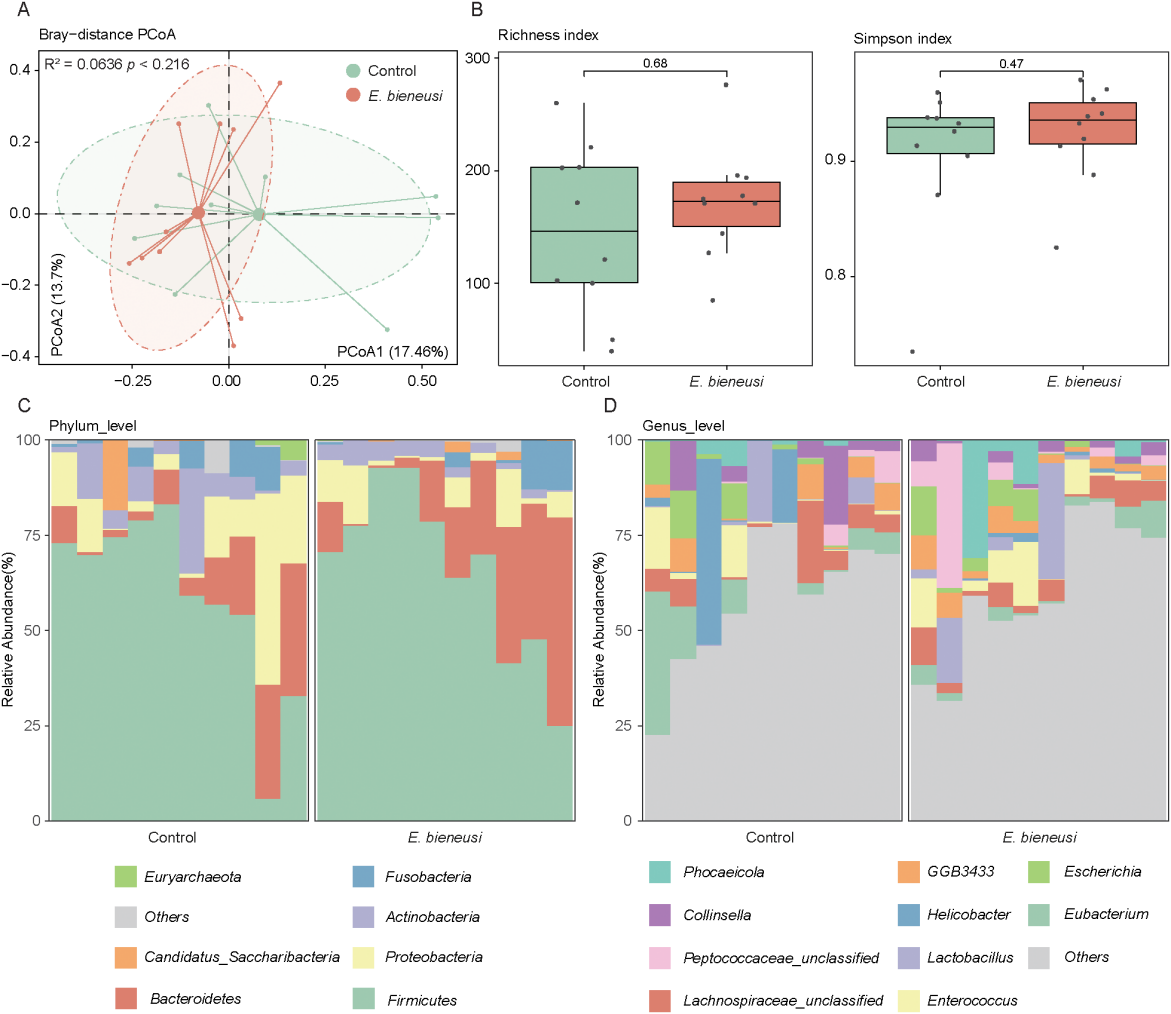
Gut microbiota diversity and composition analysis across *E*. *bieneusi* and control groups. **(A)**. The scatter plot illustrates the results of the principal coordinate analysis (PCoA) conducted on the Bray-Curtis distances for the gut microbiota across all samples. Each sample is distributed along the first and second principal coordinates (PCoA1 and PCoA2), with the associated percentage of variance accounted for displayed. The ellipsoids signify the 75% confidence intervals for each group. **(B)**. The bar chart shows the richness diversity index (left) and simpson index (right) of the gut microbiota in the *E*. *bieneusi* and control groups. **(C)**. The bar chart illustrates the community composition of the gut microbiota at the phylum level for all samples. **(D)**. The bar chart illustrates the community composition of the gut microbiota at the genus level for all samples.

Among the 20 samples, 10 were infected with *E. bieneusi* and 10 were uninfected, with no samples testing positive for C*ryptosporidium* spp., *Blastocystis*, *Giardia*, *Cyclospora* or *Balantioides coli*. To examine compositional differences in the gut microbiota between *E. bieneusi*-infected and uninfected wild mice, PCoA was performed to evaluate *E. bieneusi* and control groups at the species level. The first two principal coordinates collectively explained 31.16% of the total variance. The results revealed no clear separation between the two groups, which was further confirmed by the PERMANOVA analysis (R² = 0.0636, *P* > 0.05, **Figure 1A**). Additionally, alpha diversity was evaluated using richness and the simpson index. *P*-values from Wilcoxon rank-sum tests across comparisons were combined using Fisher’s method. The findings demonstrated no significant differences in either richness or simpson index between the two groups (*P* > 0.05, **Figure 1B**). These results indicate that *E. bieneusi* infection does not drastically alter the overall structure or diversity of the bacterial community.

The gut microbiota at different taxonomic levels were analyzed to explore the microbial structure in the gut of both healthy and diseased wild mice. At the phylum level, *Firmicutes* (62.47 ± 22.60% across all samples) and *Bacteroidetes* (16.42 ± 15.26%) were the predominant phyla, followed by *Proteobacteria* (9.87 ± 11.65%), *Actinobacteria* (5.56 ± 6.10%), and *Fusobacteria* (3.28 ± 4.76%) (**Figure 1C**, **Supplementary Table 1**). At the genus level, the most dominant taxa included *Eubacterium* (5.28 ± 8.51%), *Lachnospiraceae_unclassified* (4.93 ± 4.87%), and *Enterococcus* (4.31 ± 6.03%), followed by *Lactobacillus* (4.24 ± 8.50%), *Helicobacter* (3.89 ± 11.37%), and *GGB3433* (3.70 ± 3.18%) (**Figure 1D**, **Supplementary Table 1**).

### Virome profiling and taxonomic annotation of wild mice gut microbiota

**Figure 2:**
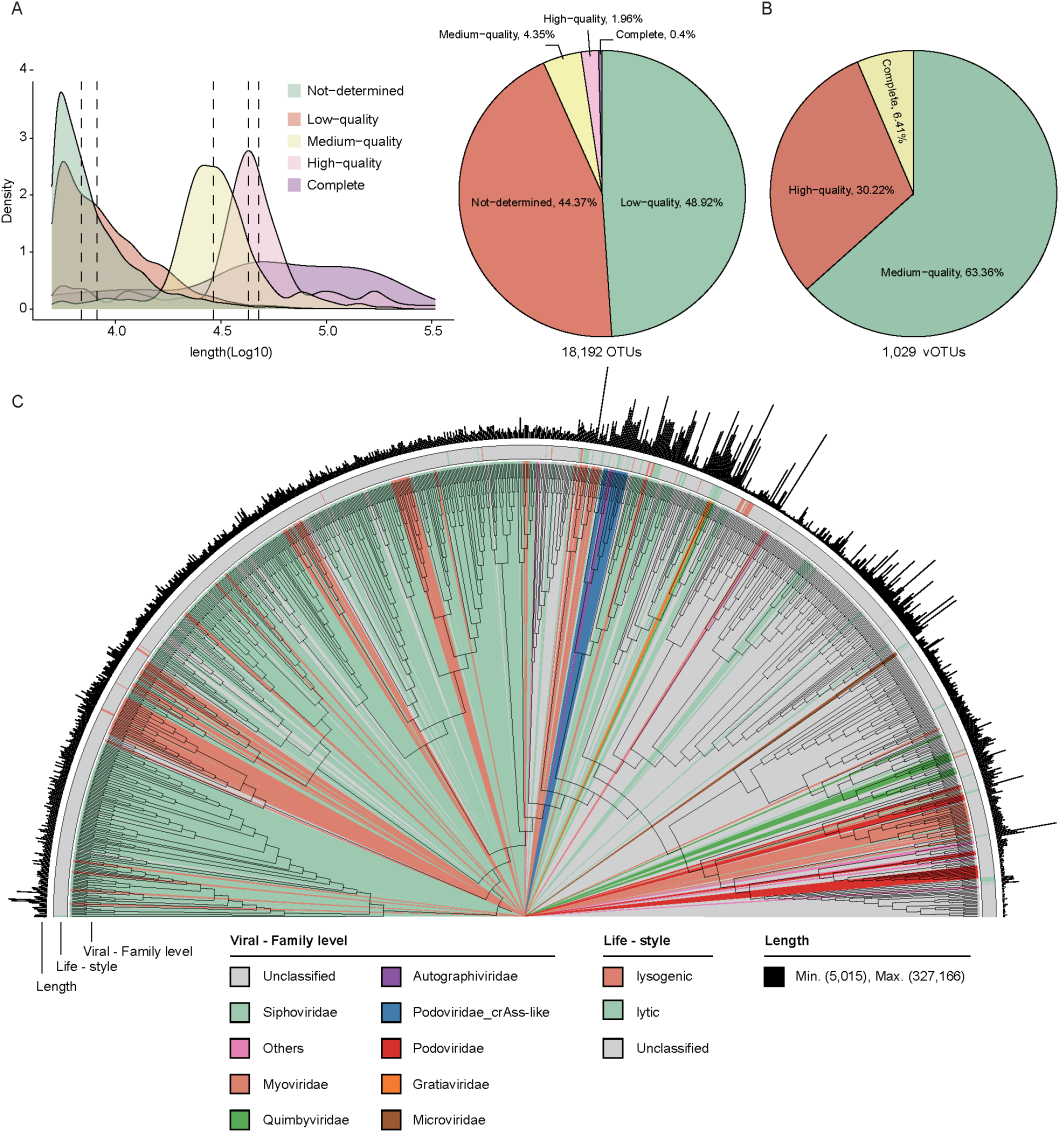
Viral genome prediction, clustering, and phylogenetic analysis. **(A)**. The density plot illustrates the distribution of viral genomes in terms of density and quantity across different quality (left). The pie chart shows the proportion of viruses in each quality. **(B)**. The pie chart displays the distribution of vOTU quality. **(C)**. The phylogenetic relationship of 1,029 vOTUs. The color coding of each evolutionary branch corresponds to its family-level classification. The first outer ring represents the life-style of the genomes, and the second outer ring indicates the genome length.

Using an integrated pipeline based on both homology and feature-based approaches (as described in Methods), we identified 18,192 highly reliable viral sequences from the 20 sample contigs. The viral sequence lengths ranged from 5,000 to 327,166 bp, with a mean length of 12,142 bp. The genome completeness and contamination were evaluated using CheckV, with 0.4% complete, 1.96% high completeness, and 4.35% medium completeness (**Figure 2A**, **Supplementary Table 2**). Next, viral sequences were clustered at a 95% nucleotide identity threshold (≥85% coverage), producing a non-redundant viral catalog containing 1,029 vOTUs. Of these, 6.41% contained a complete representative virus, while 30.22% and 63.36% had representative viruses with high and medium completeness, respectively (**Figure 2B**, **Supplementary Table 3**).

Among these non-redundant vOTUs, the viral sequence lengths ranged from 5,015 to 327,166 bp, with a mean length of 42,339 bp. Of these, 25.2% (23,594/93,462) were assigned to known viral families, covering a total of 14 families. The majority of these assigned vOTUs were classified into several families, including *Siphoviridae* (39.9%, 411/1029) and *Myoviridae* (10.4%, 107/1029). The lifestyle of 70 vOTUs was predicted, and 67.14% (47/70) classified as lytic phages (**Figure 2C**, **Supplementary Table 3**).

### Gut viral structure and diversity in wild mice infected with *E. bieneusi*

**Figure 3:**
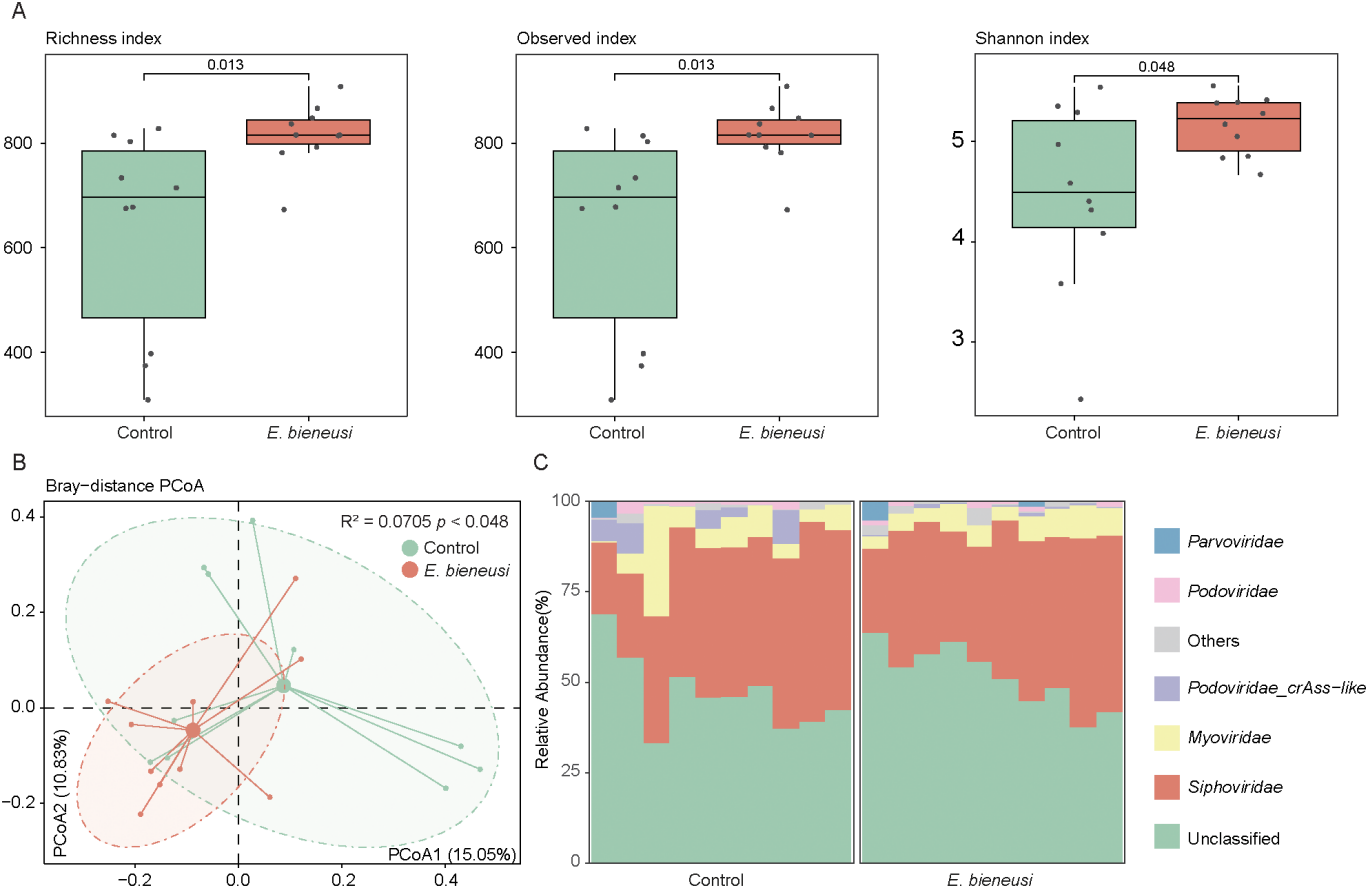
Gut virome diversity and composition analysis across *E*. *bieneusi* and control groups. **(A)**. The bar chart shows the richness diversity index (left), observed index (medium) and simpson index (right) of the gut virome for the *E*. *bieneusi* and control groups. **(B)**. The scatter plot illustrates the results of the principal coordinate analysis (PCoA) conducted on the Bray-Curtis distances for the gut virome across all samples. Each sample is distributed along the first and second principal coordinates (PCoA1 and PCoA2), with the associated percentage of variance accounted for displayed. The ellipsoids signify the 75% confidence intervals for each group **(C)**. The bar chart illustrates the community composition of the gut virome at the family level for all samples.

The clean reads were compared to the viral catalog to assess relative abundance at the family level, focusing on the diversity of the wild mice gut virome. The richness index, simpson index, and shannon index were used to assess the alpha diversity of the gut virome. Overall, wild mice infected with *E. bieneusi* showed a significantly higher diversity index in the gut virome than the control group (*P* < 0.05, **Figure 3A**). PCoA showed that PC1 and PC2 accounted for 15.05% and 10.83% of the variation, respectively, revealing significant differences in the gut virome (R² = 0.0705, *P* < 0.05, **Figure 3B**).

Known viral families represented 50.64% of the total gut virome abundance at the family level, with *Siphoviridae* (39.26 ± 9.73%) and *Myoviridae* (6.99 ± 5.95%) as the dominant families, followed by *Podoviridae_crAss-like* (1.80 ± 2.96%), *Podoviridae* (0.90 ± 0.89%) and *Parvoviridae* (0.57 ± 1.50%) (**Figure 3C**, **Supplementary Table 4**).

### Identification of viral signatures and the correlation with bacterial features

**Figure 4:**
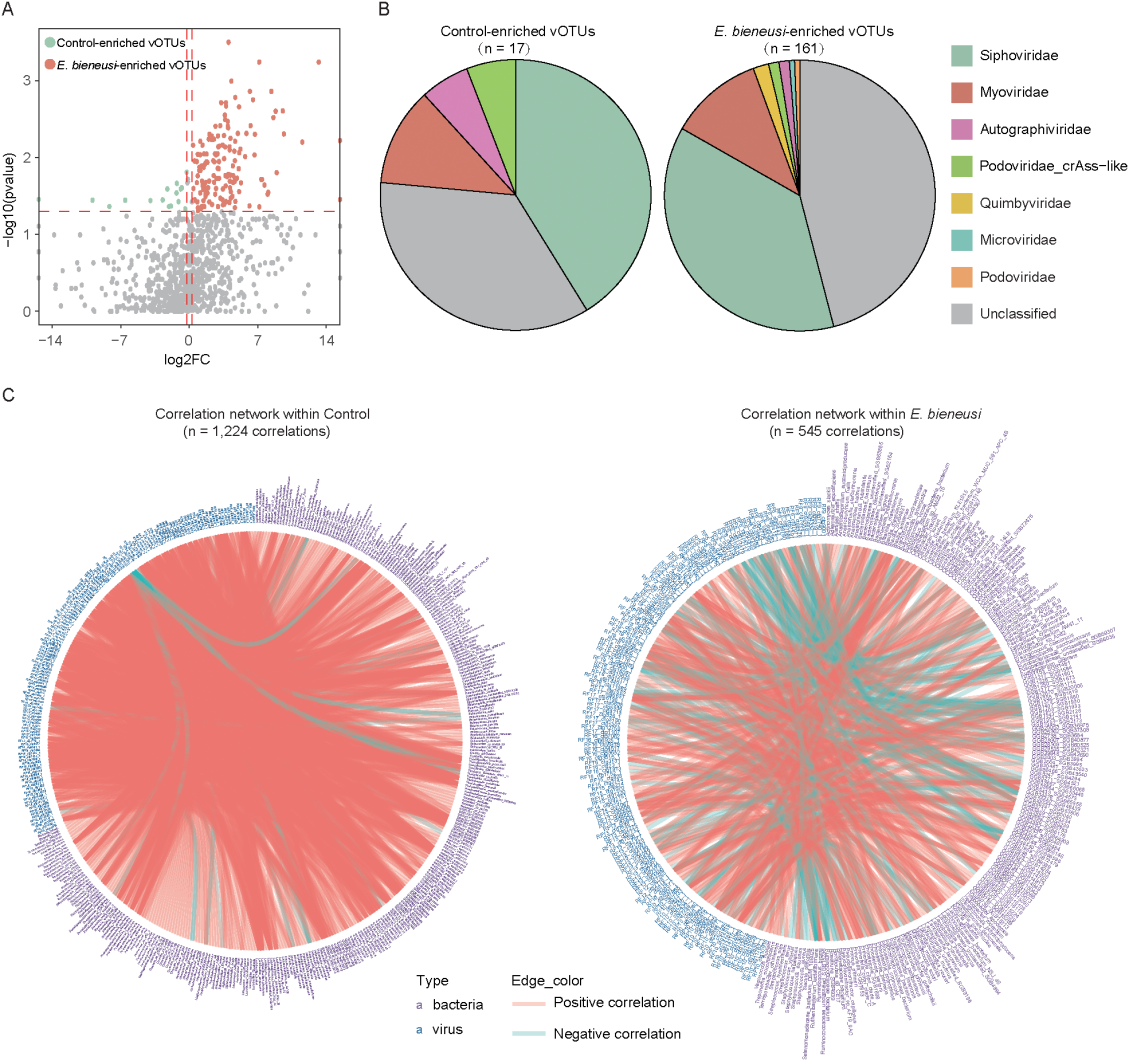
Correlation analysis between gut viral signatures and bacterial species in wild mice infected with *E*. *bieneusi*. **(A)**. The volcano plot shows the fold change and *p*-values for all vOTUs. vOTUs with an absolute fold change greater than 1.2 and *p*-values less than 0.05 are represented by red and green dots in the plot, corresponding to the *E*. *bieneusi* and control groups. **(B)**. Taxonomic distribution of control-enriched and *E*. *bieneusi*-enriched vOTUs. **(C)**. The network shows the virus-bacterium correlations in the control (left) and *E*. *bieneusi* groups (right).

To identify viral taxa in wild mice infected with *E. bieneusi*, we compared the vOTU composition between the *E. bieneusi* and control group. This analysis identified 178 vOTUs that differed significantly between groups (Wilcoxon rank-sum test, *P* < 0.05, Fold Change > 1.2, **Figure 4A**, **Supplementary Table 5**). Of these, 161 vOTUs were enriched in the *E. bieneusi* group and 17 in the control group, implying that these vOTUs might be viral taxa associated with *E. bieneusi*. Additionally, when performing viral family annotation on these vOTUs, we found a higher proportion of unclassified members in both the *E. bieneusi* and control groups (35.29% and 45.96%). The vOTUs enriched in the control group were primarily *Siphoviridae* (41.18%, 7/17). Similarly, a substantial proportion of the vOTUs enriched in the *E. bieneusi* group belonged to the *Siphoviridae* (37.27%, 60/161) and *Myoviridae* (11.18%, 18/161) (**Figure 4B**).

To explore the intricate relationship between the gut virome and bacteriome in wild mice infected with *E. bieneusi*, we performed a Spearman correlation analysis between 178 *E. bieneusi*-associated vOTUs and bacteria across two groups. Two separate correlation networks were constructed for each group, with 545 correlations identified within the *E. bieneusi* network and 1,224 correlations in the control network (Spearman’s correlation coefficient >0.8, *P* < 0.05, **Figure 4C**, **Supplementary Table 6**). Interestingly, only a small proportion of the virus-bacteria correlations (2.53%, 31 out of 1,224) in the control network were also present in the *E. bieneusi* network, suggesting that *E. bieneusi* infection leads to changes in most of the virus-bacteria associations. However, several virus-bacteria correlations were unique to the *E. bieneusi* group. For instance, *GGB3717_SGB5040*, *Fusobacteriaceae_unclassified_SGB59307* and *Barnesiella_intestinihominis* were associated with 9, 6, and 6 vOTUs, respectively, which were not detected in the control group. These results demonstrated that *E. bieneusi* infection not only reshapes virome and bacteriome composition but also restructures their ecological interactions.

### Functional alteration of the gut microbiome in wild mice infected with *E. bieneusi*

**Figure 5:**
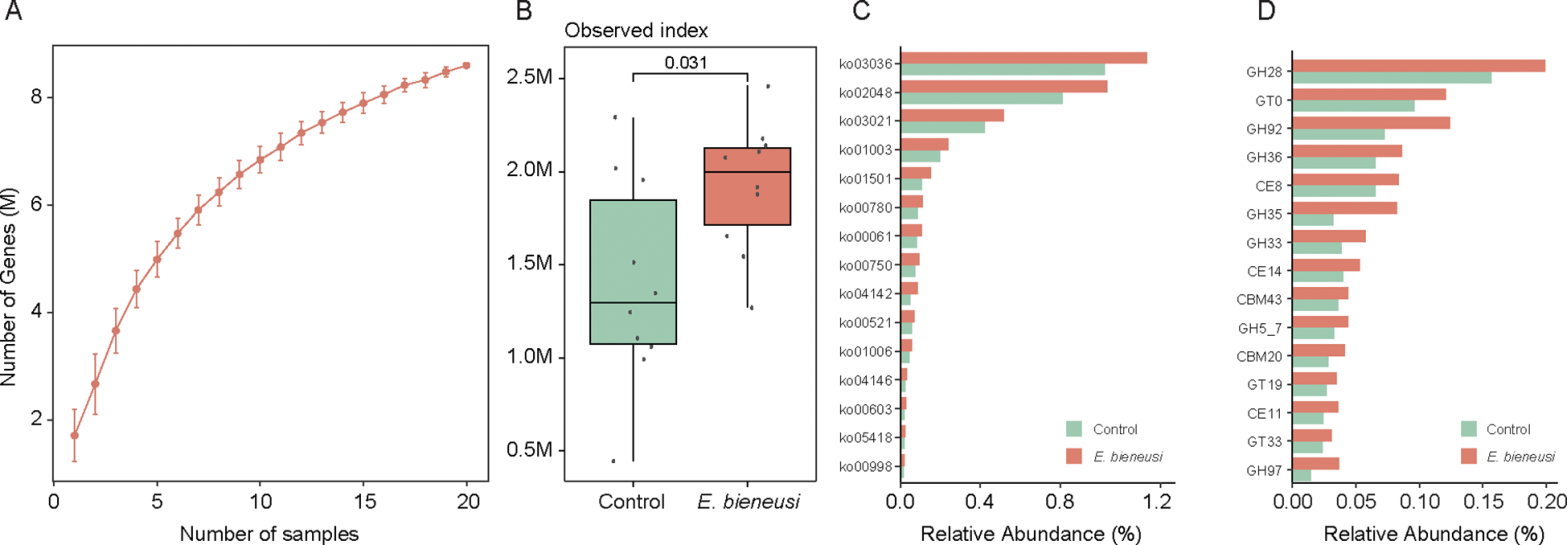
Construction of the gene catalog and comparison of microbial functions. **(A)**. Rarefaction curve of gene counts in the gene catalog. **(B)**. Comparison of microbial gene counts between the *E*. *bieneusi* and control groups. **(C)**. The bar chart shows the relative abundance of the top 15 significantly different KEGG pathways (*p* < 0.05). **(D)**. The bar chart shows the relative abundance of the top 15 significantly different CAZY families (*p* < 0.05).

To investigate the microbial functions in wild mice infected with *E. bieneusi*, we constructed a gene catalog for wild mice and analyzed the gene relative abundances. The rarefaction curve approached a saturation plateau, indicating that the sequencing depth was adequate to capture the majority of microbial genes (**Figure 5A**). Notably, the number of genes was significantly greater in the *E. bieneusi* group than in the control group (**Figure 5B**). Among the 14,682 KOs, 564 KOs showed significant differences between the two groups (*P* < 0.05). Additionally, 34 KEGG pathways also showed significant differences (**Supplementary Table 7**). We found that three pathways had higher abundance in the control group, including biosynthesis of other secondary metabolites, substance dependence, and viral protein families. In contrast, 30 pathways were significantly enriched in the *E. bieneusi* group, primarily including metabolism of terpenoids and polyketides, digestive system, biosynthesis of other secondary metabolites and metabolism of cofactors and vitamins (**Figure 5C**, **Supplementary Table 7**). Interestingly, the significant enrichment of metabolic pathways in the gut microbiota of wild mice infected with *E. bieneusi* may point to adaptive microbiome restructuring in response to infection. The enrichment of these metabolic pathways could be associated with mechanisms by which the host attempts to maintain or restore normal physiological functions during the infection process. In the CAZy family, 101 enzymes were significantly enriched in the *E. bieneusi* group (**Figure 5D**, **Supplementary Table 8**).

## Discussion

This study explores the effects of *E. bieneusi* infection on the gut microbiota and virome in wild mice. By utilizing metagenomic sequencing, we have uncovered substantial changes in both microbial composition and functional pathways, highlighting the intricate host-pathogen-microbiome interactions. Our findings have significant ecological and public health implications, especially in understanding the broader effects of parasitic infections on wildlife and their potential to affect human and livestock populations.

In this study, the mice gut microbiota was predominantly composed of the phyla *Firmicutes* and *Bacteroidetes*, which aligns with previous studies (Meng et al., 2023). Alpha diversity analysis revealed no significant differences between the *E. bieneusi* and control groups, indicating that infection may not significantly affect the overall microbial diversity. Nonetheless, several bacterial genera—such as *Lactobacillus* and *Enterococcus*, which are associated with immune regulation and ecological stability (Ingham et al., 2021; Dong et al., 2022). These results suggest that although the overall diversity remained stable, *E. bieneusi* infection may modulate the composition of specific taxa within the gut microbial community.

The diversity and composition of the gut virome showed significant changes, with *Siphoviridae* and *Myoviridae* families predominating. These viruses are known to interact with the gut bacteria (Cao et al., 2022). Additionally, previous studies have highlighted that pathogen infections can alter gut virome homeostasis, resulting in significant shifts in viral populations, thereby affecting microbial dynamics and host immune responses (Kapusinszky et al., 2012; Zuo et al., 2018). The relationship between the virome, bacteriome, and host immune response is complex and likely intertwined. Previous studies have shown that the host’s immune response to pathogens can indirectly shape the gut virome, particularly through immune signaling pathways such as the production of interferons (IFNs) and interleukins (ILs). The infection-induced immune response may lead to inflammatory conditions that promote the lytic cycle of temperate phages, further affecting microbial dynamics. Moreover, the activation of certain immune pathways could potentially modulate the abundance of specific viruses, shaping the overall virome composition and influencing the bacteriome’s structure and function (Cao et al., 2022). A key finding of this study is the identification of 178 differentially abundant vOTUs between the *E. bieneusi* and control groups. Interestingly, most of these vOTUs were enriched in the *E. bieneusi* group. Notably, it is worth noting that several virus-bacteria associations were unique to the *E. bieneusi* group. For example, virus-bacteria associations such as *GGB3717_SGB5040* and *Fusobacteriaceae*_unclassified_SGB59307 were observed, suggesting that the presence of *E. bieneusi* could drive gut microbiome interactions, potentially influencing disease outcomes.

The analysis of gut microbiome functionality indicated that infection with *E. bieneusi* resulted in considerable changes across various metabolic pathways. In particular, the pathways associated with the biosynthesis of secondary metabolites, metabolism of cofactors and vitamins, as well as the functions of the digestive system were notably enriched in the *E. bieneusi* group, suggesting adaptive metabolic adjustments of the host under infection stress (Becattini et al., 2021). The enrichment of these pathways might reflect the host’s attempt to compensate for the infection, potentially facilitating recovery or modulating immune responses. However, these functional enrichments also reflect a dysbiotic state of the gut microbiome, where the infection disrupts the normal metabolic balance of the gut microbiome. Similar findings have been reported in *Toxoplasma gondii* infections, where metabolic pathways involved in nutrient metabolism are activated (Partida-Rodríguez et al., 2017; Lu et al., 2021; Meng et al., 2023).

This study provides valuable insights into the complex interactions between the gut bacteria and gut virome in wild mice infected with *E. bieneusi*. However, there are some limitations. First, the limited sample size may restrict the generalizability of our results. Moreover, although our focus was on microbial diversity and functional pathways, the specific mechanisms driving the observed changes in the virome and bacteria are still unknown. Future studies could explore the functional roles of specific bacterial taxa and viral communities in *E. bieneusi* infection. Observing the gut microbiota before, during, and after infection through longitudinal studies would shed light on the temporal dynamics of microbiota shifts.

## Conclusion

This study provides a comprehensive analysis of the gut microbiota and virome in wild mice infected with *E. bieneusi*. Our findings demonstrate that while the infection does not significantly alter the diversity of the gut microbiota, it induces notable shifts in the viral community. The identification of 178 differentially abundant vOTUs highlights the potential for viral signatures as biomarkers of infection. Moreover, the analysis revealed that *E. bieneusi* infection induces adaptive metabolic shifts in the microbiome, which may represent the host’s mechanism for maintaining homeostasis under infection stress.

## Data Availability

A total of 20 wild mice (Rattus flavipectus) metagenomic samples are available at the National Center for Biotechnology Information (NCBI) under the project accession number PRJNA1175865.
